# CSF Amyloid and Tau Biomarkers Distinguish Mixed from Vascular Dementia by Identifying Alzheimer’s Disease Co-Pathology

**DOI:** 10.3390/medicina62050833

**Published:** 2026-04-27

**Authors:** Zuzana André, Andrea Kopániová, Barbora Gaštanová, Petra Brandoburová, Veronika Režnáková, Martin Fabian, Pavol Povinec, Jozef Hanes, Karin Gmitterová

**Affiliations:** 1 2nd Department of Neurology, Faculty of Medicine, Comenius University Bratislava, University Hospital Bratislava, 831 01 Bratislava, Slovakia; andreakopaniova@gmail.com (A.K.); gastanova7@uniba.sk (B.G.); 2 Department of Psychology, Faculty of Arts, Comenius University in Bratislava, 811 02 Bratislava, Slovakia; petra.brandoburova@uniba.sk; 3 MEMORY Centre, 851 03 Bratislava, Slovakia; veronika.reznakova@gmail.com; 4 Department of Magnetic Resonance Imaging, Dr. Magnet Ltd., 831 01 Bratislava, Slovakia; mfabian@prodiagnostic.sk; 5 PET/CT Centre, Biont a.s., 842 29 Bratislava, Slovakia; povinec@biont.sk; 6 Slovak Academy of Sciences, Institute of Neuroimmunology, 841 04 Bratislava, Slovakia; jozef.hanes@savba.sk; 7 Department of Neurology, Faculty of Medicine, Slovak Medical University, University Hospital Bratislava, 821 01 Bratislava, Slovakia

**Keywords:** Alzheimer’s disease, vascular dementia, mixed dementia, CSF biomarkers, differential diagnosis

## Abstract

*Background and Objectives:* Vascular dementia (VaD) and mixed dementia (MD) represent prevalent causes of cognitive decline in the elderly, as they share similar pathological pathways and clinical features. Distinguishing between these two conditions remains a challenge, due to their frequent clinical and neuroimaging overlap. Nevertheless, it is important from a prognostic perspective. *Materials and Methods:* The study comprised 114 participants, including patients with VaD (*n* = 33), MD (*n* = 26), Alzheimer’s disease (AD; *n* = 26), and 29 cognitively healthy controls (C). We evaluated routinely used cerebrospinal fluid (CSF) biomarkers (total tau, p-tau181, Aβ_1–42_) and their ratios to assess inter-group differences, diagnostic accuracy, and correlations with cognitive score. *Results:* Patients with MD demonstrated significantly higher levels of t-tau and p-tau181, and lower levels of Aβ_1–42_, compared to VaD (*p* < 0.004 for all analyses). With the exception of p-tau181/t-tau, all calculated ratios enabled differentiation between these groups. ROC analysis confirmed the high diagnostic accuracy of CSF Aβ_1–42_ and t-tau (AUC 0.82 and 0.79 respectively) for detecting AD pathology in dementia patients. Furthermore, the t-tau/Aβ_1–42_, p-tau181/Aβ_1–42_ ratios were the most effective in differentiating AD-related from vascular pathologies (AUC 0.78 and 0.80 respectively), and in differentiating MD from VaD (AUC 0.79 and 0.77 respectively). A significant correlation was observed between CSF biomarkers (especially tau markers) and cognitive impairment severity. *Conclusions*: CSF biomarkers effectively differentiate mixed from vascular dementia by identifying underlying AD pathology independent of the clinical phenotype. This supports the use of CSF biomarkers in clinical practice to reveal the neurodegenerative component in patients with cerebrovascular disease, which is of fundamental importance for emerging disease-modifying treatment strategies in mixed neuropathologies.

## 1. Introduction

Alzheimer’s disease (AD) and vascular dementia (VaD) represent the most prevalent causes of cognitive decline in the elderly population. Alzheimer’s disease is characterized by extracellular accumulation of amyloid beta (Aβ) plaques and intracellularly located neurofibrillary tangles consisting of the aggregates of hyperphosphorylated tau forms (p-tau) [1], whereas VaD is predominantly driven by subcortical small vessel disease (SVD) [2]. Cerebrovascular injury becomes increasingly prevalent with age, rising from 5% in 50-year-old individuals to 90% in those over 90 [3]. In age-related cognitive decline, vascular and degenerative pathologies frequently co-occur and potentiate neurodegenerative processes [4], having an additive effect on cognitive decline [5]. Vascular dysfunction, impaired amyloid clearance, and neuroinflammation are responsible for the synergistic effects of these pathologies in neurodegeneration [6].

The interplay between vascular and Alzheimer’s pathologies is viewed as a vicious cycle. While vascular injury from SVD promotes the formation of amyloid plaques and tau tangles, the presence of Aβ simultaneously erodes the structural integrity of the neurovascular unit [7]. This dual assault weakens the blood–brain barrier, allowing neurotoxic molecules to leak into the brain tissue and lead to neuroinflammation. Subsequent axonal and white matter damage acts as a primary catalyst for cognitive decline, highlighting the inseparable nature of vascular and proteinopathic brain aging [8]. Furthermore, some cerebrovascular risk factors, like midlife hypertension and diabetes, also increase AD risk [9].

This overlap presents a significant diagnostic challenge, as the clinical diagnosis of AD lacks specificity, which is estimated at approximately 48% [10]. Therefore, imaging and cerebrospinal fluid (CSF) biomarkers are essential for improving diagnostic accuracy in AD. Common CSF markers, specifically Aβ_1–42_, total tau, and p-tau181, are well-validated and widely accepted [11,12]. Along with amyloid and tau PET/CT imaging and MRI-detected atrophy, these biomarkers are integral to the Amyloid/Tau/Neurodegeneration (ATN) classification system [13], which redefines AD as a biological construct, rather than merely a clinical syndrome.

However, shared underlying pathologies can limit diagnostic certainty, despite the availability of biomarker evidence. For instance, approximately 30% of patients with subcortical VaD test false positive on amyloid PET scans [14]. While the combination of at least two CSF biomarkers yields higher sensitivity and specificity for differentiating AD from healthy controls [15], their utility in differential diagnosis against other dementias is more complex. Previous studies indicate that the diagnostic accuracy of these biomarkers decreases in mixed clinical cohorts, as both CSF and amyloid PET profiles in AD can substantially overlap with those seen in other forms of dementia, including VaD [16,17,18]. Quantifying cortical atrophy via MRI and assessing dual pathology in SVD can be misleading because cortical thinning is not solely a feature of AD-like degeneration; it is also driven by SVD mechanisms. Notably, white matter hyperintensities (WMHs) disrupt subcortical white matter tracts, leading to axonal damage and secondary cortical degeneration, while concurrent SVD-associated hypoperfusion and hypometabolism further exacerbate this atrophy [19,20].

Such co-occurrence underscores the limitations of using biomarkers to differentiate common vascular injury from AD in the elderly [14]. Diagnostic criteria for mixed dementia remain undefined due to pathological overlap, insufficient vascular biomarkers, and the difficulty of distinguishing the predominant pathology in vivo. The main aim of our study was to evaluate the diagnostic potential of routinely measured CSF biomarkers in confirming VaD diagnosis versus mixed (AD and vascular) dementia.

## 2. Materials and Methods

### 2.1. Participants

This was a multicenter, case–control study. Patients were recruited from May 2023 to June 2025 through the memory clinic MEMORY Centre, in Bratislava, and 2nd Department of Neurology, University Hospital Bratislava, Slovakia. The diagnostic workup included a neurological examination, routine laboratory testing, and a cognitive examination by a trained clinician, where the Montreal Cognitive Assessment (MoCA) was used for stratification. A brain MRI with volumetric analysis was performed on all patients. Regarding biomarkers, patients underwent lumbar puncture focused on the dementia profile, and nine patients underwent amyloid PET imaging (all with positive result). All imaging and clinical assessments were completed, and the final diagnoses were established before the CSF biomarker results were available. CSF biomarkers, therefore, did not contribute to the diagnostic classification and were instead compared with the established clinical–radiological diagnosis as an independent benchmark.

A total of 114 participants met the inclusion criteria. The study included 33 patients with VaD who met the VICCCS 2017 criteria for major Vascular Cognitive Impairment caused by subcortical ischemic vascular dementia [21]. Patients with MD (*n* = 26) fulfilled core clinical criteria for all-cause dementia according to NIA-AA 2011 criteria [11] and had proven disproportionate atrophy in the hippocampi and/or the temporoparietal cortex by MRI volumetry (≤1st percentile in an age- and sex-matched population), along with the presence of WMH with a Fazekas score ≥ 2 on FLAIR images and/or a relevant history of vascular disorders. Additionally, a group of cognitively healthy controls was included, consisting of 29 participants who underwent lumbar puncture for various indications (depression, headache, and fatigue). Eligibility for the cognitively healthy control group required the absence of subjective cognitive impairment [22]. In addition to VaD, MD, and healthy controls, a group of patients with a clinical diagnosis of probable AD (*n* = 26) was included as a reference group. AD patients were classified according the NIA-AA 2011 criteria [11], ensuring consistency with the AD process through typical findings (hippocampal atrophy) detected on MRI volumetry and a Fazekas score ≤ 1 on FLAIR images. Nine patients with AD underwent PET imaging and proved amyloid positivity. Exclusion criteria for all patient subgroups were: age under 55 years, evidence of an inflammatory neurological disorder based on imaging and/or CSF results, cognitive impairment caused by other conditions such as psychiatric disease, systemic and/or metabolic/autoimmune disorders, parkinsonism, prominent behavioral or language disorders early in the course of disease, rapid cognitive decline (within < 12 weeks), and history of stroke within the last three months. This study was approved on April 4 2023 by the local Ethics Committee of the Bratislava Self-Governing Region (6265/2023/HF) and performed in accordance with the Declaration of Helsinki. Informed consent was obtained from all patients or their caregivers.

### 2.2. Sampling and Analysis of CSF

CSF samples were obtained by lumbar puncture and processed immediately. All CSF samples were processed according to a standard protocol [23]. Routine investigation of the CSF did not reveal any abnormalities with respect to cell count, proteins, and immunoglobulins. Blood-contaminated samples were excluded from analysis. Commercial ELISA kits from Roche Diagnostics GmbH, Germany were used for analysis of total tau (t-tau), phosphorylated tau181 (p-tau181) and amyloid Aβ_1–42_. Additionally, the biomarker ratios t-tau/Aβ_1–42_ and p-tau181/Aβ_1–42_, as well as p-tau181/t-tau, were calculated.

### 2.3. Neuroradiologic Assessment

All MRI scans were acquired at Dr. Magnet (Bratislava, Slovakia) using a PHILIPS Ingenia Elition X 3.0 T. Visual evaluation of the brain was focused on assessing WMH using the Fazekas score [24]. The Standards for Reporting Vascular Changes on Neuroimaging 2 (STRIVE-2) [25] definitions and guidelines for the measurement of cerebrovascular lesions were adopted. MR angiography was performed in all patients as part of the MRI protocol. Subsequently, all MRI scans were analyzed using Icobrain DM software (Icometrix, Leuven, Belgium) for automated volumetric analysis of whole-brain volume, total hippocampal volume, and grey matter volume of the frontal, parietal, and temporal lobes. Quantification of amyloid plaques was performed with 18F-flutemetamol (Vizamyl PET).

### 2.4. Statistical Analysis

The Prism 8 program was used for statistical data processing. Descriptive statistics were calculated for every group. Significances were tested using Student’s *t*-test or the Mann–Whitney rank-sum test; for more than 2 groups, the Kruskal–Wallis test was used. Comparison of clinical, neuropsychological, and CSF biomarker characteristics between study groups was performed by ANOVA (with Bonferroni correction for multiple comparisons) or the Kruskal–Wallis test, as appropriate. In order to consider the clinical relevance of CSF biomarkers and biomarker ratios in differentiating patients with AD pathology from all other clinical groups irrespective of the clinical phenotype, AUC of Receiver Operating Characteristic (ROC) Curve analysis was performed to compare the diagnostic accuracy in differentiating patient groups. The Youden index was used to establish the optimal cut-off point. A value of *p* < 0.05 was considered significant. One Aβ_1–42_ value in the AD group was missing due to a preanalytical issue, and was therefore excluded solely from Aβ_1–42_-related analyses, whereas its t-tau and p-tau181 values were retained in the corresponding comparisons and ROC analyses.

## 3. Results

### 3.1. Patient Characteristics

Measurements of selected CSF marker levels were performed on 33 patients with VaD (16 women, 17 men), 26 patients fulfilling the criteria for MD (17 women, nine men), 26 patients diagnosed with AD (11 women, 15 men), and 29 cognitively healthy subjects (15 women, 14 men). Clinical and demographic data are displayed in Table 1.

### 3.2. CSF Markers Analysis

**Figure 1:**
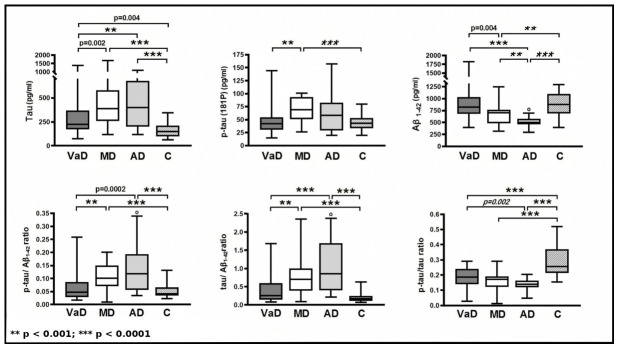
Comparison of CSF biomarker levels by diagnosis.

Significantly higher levels of t-tau (*p* = 0.002) and p-tau181 (*p* < 0.001), but lower Aβ_1–42_ CSF levels (*p* = 0.004), were demonstrated in the group of patients with MD compared to the levels in VaD. Calculated ratios p-tau/Aβ_1–42_ and t-tau/Aβ_1–42_ (*p* < 0.001 for both analyses) were additionally helpful in the differentiation between these groups. Except lower CSF Aβ_1–42_ levels in the AD (*p* < 0.001), no other clinically relevant differences in the CSF marker levels enabling distinction between AD and mixed dementia were revealed. There were significantly elevated levels of t-tau (*p* = 0.01), higher t-tau/Aβ_1–42_ (*p* < 0.0001) and p-tau181/Aβ_1–42_ (*p* = 0.002), but lower CSF Aβ_1–42_ (*p* < 0.0001) and p-tau181/t-tau (*p* = 0.002) in AD group as compared to VaD. Furthermore, all CSF markers were significantly different in MD group as compared to controls. VaD was differentiated from controls by t-tau (*p* < 0.004) and the p-tau181/t-tau ratio. Summary of results is shown in the Table 2, Figure 1.

Box plots illustrate the distributions of Aβ_1–42_, t-tau, and p-tau181 levels and their ratios across the diagnostic groups. The horizontal line within each box represents the median, the box boundaries indicate the interquartile range (25th–75th percentiles), and the whiskers represent the maximum and minimum values. Statistical significance is indicated by asterisks: ** *p* < 0.001; *** *p* < 0.0001. ° represents the one missing Aβ_1–42_ value. Abbreviations: t-tau, total tau; p-tau, phosphorylated tau181; VaD, vascular dementia; MD, mixed dementia; AD, Alzheimer’s disease; C, control.

### 3.3. Diagnostic Performance of CSF Biomarkers

In order to consider the clinical relevance of CSF biomarkers in identifying AD pathology irrespective of the clinical phenotype, we calculated the AUCs of ROC curves of various biomarkers. CSF t-tau levels confirmed potential in the distinction of AD pathology enabling the differentiation of AD/MD patients from non-AD patients (corresponding cut-off based on the Youden index was 252 pg/mL (ROC-AUC of 0.79); in the distinction from cognitively healthy controls, t-tau achieved an ROC-AUC of 0.89 (sensitivity 85% and 76% specificity; *p* < 0.0001 for both analyses). Similar results in diagnostic potential were observed for CSF Aβ_1–42_ demonstrating the ROC-AUC 0.82 (cut-off < 726 pg/mL with 85% and 71% specificity) for differentiation of AD pathology in dementia patients (*p* < 0.0001). Additionally, all calculated ratios (p-tau181/Aβ_1–42_, p-tau181/t-tau, and t-tau/Aβ_1–42_) proved diagnostic potential in the discrimination of AD-related pathology from not only non-AD causes (AUC 0.85, AUC 0.80, and AUC 0.85, respectively; *p* < 0.001 for all analyses), but vascular causes as well (AUC 0.78, AUC 0.75, and AUC 0.80); however, useful in the distinction between MD and VaD were t-tau/Aβ_1–42_, with ROC-AUC 0.79, and p-tau181/Aβ_1–42_ ratio, with AUC 0.77 (*p* < 0.001).

### 3.4. Association Between CSF Biomarkers and Cognitive Impairment

**Figure 2:**
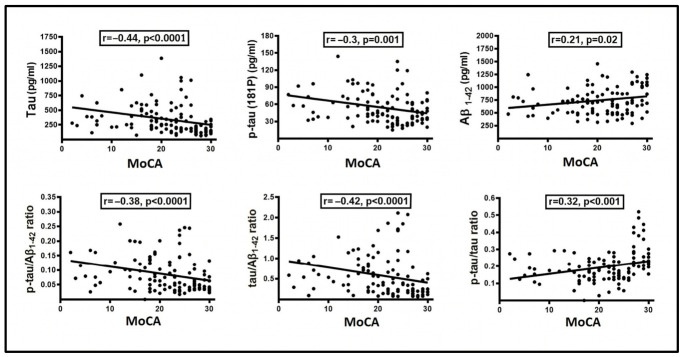
Correlation between CSF biomarkers and cognitive impairment.

We correlated level of biomarkers and complementary calculated ratios with the severity of cognitive impairment using the MoCA score. Despite the trend towards higher values of p-tau181/Aβ_1–42_ and t-tau/Aβ_1–42_ and lower p-tau181/t-tau in MD group, no significant correlation was shown in particular dementia groups. However, there was a correlation between MoCA score and level of assessed markers and calculated ratios significant for t-tau (r = −0.44, *p* < 0.0001), p-tau181 (r = −0.3, *p* = 0.001), Aβ_1–42_ (r = 0.21, *p* = 0.02), and calculated ratios p-tau181/t-tau (r = 0.32, *p* < 0.001) and p-tau181/Aβ_1–42_ (r = −0.38, *p* < 0.0001), as well as t-tau/A_β1–42_ (r = −0.42, *p* < 0.0001), when analyzing the dementia cohort as a whole (Table 1, Figure 2).

Abbreviations: t-tau, total tau; p-tau, phosphorylated tau181; MoCA, Montreal Cognitive Assessment.

## 4. Discussion

Relying solely on imaging findings to diagnose VaD often leads to overdiagnosis [27]. Dual pathology can be obscured by an overlap that is not reliably detected through clinical assessment or neuroimaging. Consequently, the primary aim of our study was to evaluate the diagnostic potential of routinely measured CSF biomarkers in differentiating “pure” VaD from mixed (vascular and AD) dementia. Our findings suggest that routine CSF biomarkers might offer diagnostic value in separating AD-related cases from predominantly vascular pathology (specifically p-tau181/Aβ_1–42_ and t-tau/Aβ_1–42_ ratios). Moreover, CSF levels of the assessed biomarkers correlated with cognitive status, with the strongest correlation observed for tau pathology.

We observed significant differences between VaD and MD in all measured CSF biomarkers and ratios, except for the p-tau181/t-tau ratio. These results confirm that, despite the role of vascular factors in potentiating AD-related pathology, CSF biomarkers are useful in the diagnosis of pure VaD. In contrast, CSF markers in patients with MD approached the values observed in pure AD, probably reflecting more pronounced tau/amyloid pathology [6]. Consistent with previous research, this indicates that the CSF profile characteristic of AD (partially also in MD) can be reliably differentiated from that of pure VaD using standard CSF panels [28,29].

Interestingly, the p-tau181/t-tau ratio did not significantly differ between the VaD and MD groups. This implies that, while the absolute levels of both markers increase in MD due to mixed pathology, they do so proportionally. Specifically, while AD pathology drives p-tau elevation, the co-existing vascular damage likely contributes additional total tau through nonspecific neuronal injury. This balanced increase in both markers effectively masks the underlying differences when expressed as a ratio, preserving a profile similar to that of VaD despite the higher absolute disease burden in MD. This “parallel” elevation can increase diagnostic uncertainty when values fall near cut-off thresholds [30,31].

In our cohort, total tau and the p-tau181/t-tau ratio were the only parameters capable of distinguishing VaD from healthy controls, thereby strengthening the value of CSF markers in excluding pseudodementia. In this regard, it is essential to emphasize that the characterization of a healthy reference group necessitates objective neuropsychological assessment without subjective cognitive complaints from participants, as SCI does not consistently offer a reliable basis for a healthy control group. While t-tau levels in pure VaD are generally lower than in AD [32], the modest elevation observed in VaD likely reflects non-specific shared axonal degeneration, rather than AD-related pathology, yet it still preserves the discriminative ability between vascular and AD-related neuropathologies [28]. Therefore, t-tau serves as a non-specific marker of neurodegeneration detectable in VaD [33,34]. Similarly, a previous study has indicated that other general neuronal injury markers (such as 14-3-3 and neuron-specific enolase) are also elevated in VaD, but these are insufficient for distinguishing VaD from AD [28].

In line with a previous study [17], we observed that p-tau alone is insufficient for distinguishing between AD and VaD. However, this distinction was successfully achieved using the p-tau181/t-tau ratio. This underscores the limitation of single biomarkers in heterogeneous cohorts, where vascular pathology may coexist with subclinical neurodegeneration. The ratio effectively standardized specific AD pathology (p-tau) against general neurodegeneration (t-tau), thereby enhancing diagnostic specificity and reducing the risk of misclassification in patients with vascular cognitive impairment.

The ratios differed significantly in all comparisons of VaD versus MD (except for p-tau181/t-tau) and versus AD. Previous research has shown that the Aβ_1–42_/t-tau ratio, or the t-tau/Aβ_1–42_ and p-tau181/Aβ_1–42_ ratios, provide the best diagnostic accuracy in distinguishing between vascular versus AD pathology. These results support the utilization of combined CSF biomarkers to effectively differentiate between these disorders [28] and underscore the importance of a multi-marker approach, as relying solely on amyloid positivity for an AD diagnosis compromises diagnostic accuracy [15]. While ratios such as p-tau181/Aβ_1–42_ and t-tau/Aβ_1–42_ improve diagnostic accuracy and better reflect the underlying AD pathology in mixed cases, clinical–biological mismatches still occur [35,36].

When comparing the MD and AD subgroups, the biomarker profiles were largely similar. Aβ_1_−_42_ was the only parameter showing a significant difference, with higher CSF levels observed in MD compared to pure AD. This finding implies a comparatively reduced amyloid burden in MD and supports the hypothesis of an “additive” effect: the presence of vascular damage may contribute to cognitive decline, allowing dementia to manifest at a lower threshold of amyloid pathology. Nevertheless, it must be noted that these differences are often reported as inconsistent and may lack sufficient robustness for clinical relevance [18,37]. The inter-individual variability of CSF biomarker values in our cohort reflects the well-recognized biological and demographic heterogeneity of dementia populations (age, sex, APOE status), driven by co-existing pathologies, disease stage, AD molecular subtypes, and differences in amyloid clearance and neurovascular integrity [38].

Regarding other CSF biomarkers for differentiating between VaD and MD, various studies point toward biomarkers associated with vascular and neuronal injury, such as the neurofilament light chain (NfL), lipocalin-2 (LCN2), and soluble amyloid precursor protein beta (sAPPβ). These can be analyzed in combination with standard CSF panels to sensitively detect both vascular and AD pathologies [39,40]. Lipocalin-2 shows particular promise as a biomarker for MD, given its involvement in both vascular and neurodegenerative processes, with levels reported to exceed those found in both pure AD and controls. Nevertheless, further research is required to confirm the clinical utility of LCN2 in this context [28,41,42].

Measured CSF biomarker levels were significantly associated with cognitive functions in the dementia cohort. Although we observed the same trends in the specific dementia groups, correlations within individual diagnostic groups did not reach statistical significance, likely due to reduced sample sizes and a limited range of cognitive scores. However, in the pooled analysis, markers of neurodegeneration (t-tau and p-tau181) showed stronger correlations with cognitive decline, compared to Aβ_1–42_. We similarly observed significant correlations for all ratios. This aligns with established evidence that, while amyloid deposition primarily mirrors early pathology and is less directly correlated with cognitive status, the tau cascade is more closely linked to the stage of neurodegeneration and the severity of clinical symptoms [37,43].

In our cohort, diagnostic groups were defined on the basis of a clinical and multimodal imaging workup (structural and volumetric MRI, and amyloid PET in a subset of patients), providing an independent imaging-based reference for assessing the diagnostic performance of the CSF biomarkers. In relation to this reference, our ROC curve analysis confirms that CSF biomarkers—particularly their ratios—serve as robust tools for identifying AD pathology. Of particular clinical importance is the ability of the t-tau/Aβ_1–42_ and p-tau181/Aβ_1–42_ ratios to satisfactorily differentiate AD from vascular pathology, and particularly MD from pure VaD.

Our study has several limitations. First, the diagnoses were mainly based on clinical criteria and neuroimaging. Although standardized criteria were strictly followed, the possibility of misclassification or the presence of mixed pathology in clinically “pure” phenotypes cannot be entirely excluded. The mixed dementia group represents a heterogeneous population with varying degrees of AD and vascular contributions, which may influence biomarker levels and complicate the interpretation of cut-off values. In addition to the relatively small subgroup sample sizes, Aβ_1−42_ analysis could not be performed for one AD patient, due to a minor preanalytical issue. A further limitation is the absence of Aβ_1–40_ measurements, preventing the calculations of the Aβ_1–42_/Aβ_1–40_ ratio, which has been shown to demonstrate superior diagnostic accuracy compared to Aβ_1–42_ alone [44,45]. Due to the study design and the relatively small sample size of a specific subgroup, the ROC analysis was conducted on the entire cohort, lacking an independent validation cohort. Despite promising results, further validation in larger studies to ensure clinical reliability is required.

## 5. Conclusions

In conclusion, CSF biomarkers and their calculated ratios enable accurate identification of AD pathology independent of clinical phenotype, effectively distinguishing mixed dementia from pure vascular dementia. Given the strong correlation with MoCA scores, these markers represent a reliable tool for clinical practice in determining the etiology of cognitive impairment. This supports the use of CSF biomarkers in clinical practice to reveal the neurodegenerative component in patients with cerebrovascular disease, which is of fundamental importance not only from a prognostic aspect, but also for emerging disease-modifying treatment strategies.

## Figures and Tables

**Table 1:** Clinical and demographic characteristics of the study participants (*n* = 114).

	VaD	MD	AD	Controls
*n*, male/female	33 (17/16)	26 (9/17)	26 (15/11)	29 (14/15)
Age at onset (median/range)	76 (58–88)	77 (67–87)	72 (55–78)	69 (56–79)
Time from diagnosis to LP, months (median, range)	13 (4–23)	9 (3–20)	11 (2–16)	
Cognitive status (MoCA) ^†^ (mean/± SD)	18.8 (±5.6)	15.5 (±6.0)	23 (±3.4)	28.5 (±1.3)
Years of education	12 (±1.8)	14 (±2.1)	13 (±2.2)	15 (±1.6)

^†^ For five participants, MoCA scores were calculated from MMSE according to the conversion table [26]. Abbreviations: SD, standard deviation; VaD, vascular dementia; MD, mixed dementia; AD, Alzheimer’s disease; LP: lumbar puncture; MoCA, Montreal Cognitive Assessment.

**Table 2:** Levels of investigated CSF biomarkers and calculated ratios.

	VaD	MD	AD	C
t-tau (pg/mL) (median/range)	224 (74–1386)	388 (117–1671)	401 (119–1100)	151 (64–341)
p-tau181 (pg/mL) (median/range)	42 (15–144)	69 (26–101)	58 (24–157)	43 (20–80)
Amyloid β_1–42_ (pg/mL) (median/range) *	821 (395–1828)	701.5 (318–1244)	500 (295–698) *	874 (401–1295)
p-tau/t-tau ratio (median/range)	0.188 (0.028–0.292)	0.172 (0.02–0.292)	0.140 (0.05–0.21)	0.256 (0.154–0.52)
p-tau/Aβ_1–42_ ratio (median/range) *	0.048 (0.017–0.258)	0.101 (0.013–0.201)	0.118 (0.035–0.339) *	0.040 (0.023–0.132)
t-tau/Aβ_1–42_ ratio (median/range) *	0.260 (0.07–1.688)	0.703 (0.09–2.354)	0.854 (0.211–2.376) *	0.179 (0.067–0.625)

* One value is missing. Abbreviations: t-tau, total tau; p-tau, phosphorylated tau181; VaD, vascular dementia; MD, mixed dementia; AD, Alzheimer’s disease; C, control.

## Data Availability

The original contributions presented in this study are included in the article; further inquiries can be directed to the corresponding authors.

## Abbreviations

The following abbreviations are used in this manuscript:

CSF	Cerebrospinal Fluid
VaD	Vascular dementia
MD	Mixed dementia
AD	Alzheimer’s disease
C	Controls
t-tau	Total tau
p-tau	Phosphorylated tau
MR	Magnetic Resonance
VICCCS	The Vascular Impairment of Cognition Classification Consensus Study
NIA-AA	National Institute on Aging and Alzheimer’s Association
ROC	Receiver Operating Characteristic
AUC	Area Under the Curve
Aβ	Amyloid beta
PET/CT	Positron Emission Tomography/Computed Tomography
WMH	White matter hyperintensity
SVD	Small vessel disease
MoCA	Montreal Cognitive Assessment
LP	Lumbar puncture

## References

[1] Scheltens P., De Strooper B., Kivipelto M., Holstege H., Chételat G., Teunissen C.E., Cummings J., van der Flier W.M. (2021). Alzheimer’s Disease. Lancet.

[2] Wardlaw J.M., Smith C., Dichgans M. (2019). Small Vessel Disease: Mechanisms and Clinical Implications. Lancet Neurol..

[3] Cannistraro R.J., Badi M., Eidelman B.H., Dickson D.W., Middlebrooks E.H., Meschia J.F. (2019). CNS Small Vessel Disease: A Clinical Review. Neurology.

[4] de la Torre J.C. (2002). Vascular Basis of Alzheimer’s Pathogenesis. Ann. N. Y Acad. Sci..

[5] Snowdon D.A., Greiner L.H., Mortimer J.A., Riley K.P., Greiner P.A., Markesbery W.R. (1997). Brain Infarction and the Clinical Expression of Alzheimer Disease. The Nun Study. JAMA.

[6] Sachdev P.S., Bentvelzen A.C., Gustafson D., Hansra G.K., Hosoki S., Jiang J., Lennon M.J., Moro M.A., Saks D.G., Samaras K. (2026). Vascular Cognitive Impairment and Dementia: Clinical Features, Neuropathology, and Biomarkers. J. Am. Coll. Cardiol..

[7] Kim H.W., Hong J., Jeon J.C. (2020). Cerebral Small Vessel Disease and Alzheimer’s Disease: A Review. Front. Neurol..

[8] Fisher R.A., Miners J.S., Love S. (2022). Pathological Changes within the Cerebral Vasculature in Alzheimer’s Disease: New Perspectives. Brain Pathol..

[9] Chui H.C., Zheng L., Reed B.R., Vinters H.V., Mack W.J. (2012). Vascular Risk Factors and Alzheimer’s Disease: Are These Risk Factors for Plaques and Tangles or for Concomitant Vascular Pathology That Increases the Likelihood of Dementia? An Evidence-Based Review. Alzheimers Res. Ther..

[10] Knopman D.S., DeKosky S.T., Cummings J.L., Chui H., Corey-Bloom J., Relkin N., Small G.W., Miller B., Stevens J.C. (2001). Practice Parameter: Diagnosis of Dementia (an Evidence-Based Review). Report of the Quality Standards Subcommittee of the American Academy of Neurology. Neurology.

[11] McKhann G.M., Knopman D.S., Chertkow H., Hyman B.T., Jack C.R., Kawas C.H., Klunk W.E., Koroshetz W.J., Manly J.J., Mayeux R. (2011). The Diagnosis of Dementia Due to Alzheimer’s Disease: Recommendations from the National Institute on Aging-Alzheimer’s Association Workgroups on Diagnostic Guidelines for Alzheimer’s Disease. Alzheimers Dement..

[12] Dubois B., Feldman H.H., Jacova C., Hampel H., Molinuevo J.L., Blennow K., DeKosky S.T., Gauthier S., Selkoe D., Bateman R. (2014). Advancing Research Diagnostic Criteria for Alzheimer’s Disease: The IWG-2 Criteria. Lancet Neurol..

[13] Jack C.R., Andrews J.S., Beach T.G., Buracchio T., Dunn B., Graf A., Hansson O., Ho C., Jagust W., McDade E. (2024). Revised Criteria for Diagnosis and Staging of Alzheimer’s Disease: Alzheimer’s Association Workgroup. Alzheimers Dement..

[14] Lee J.H., Kim S.H., Kim G.H., Seo S.W., Park H.K., Oh S.J., Kim J.S., Cheong H.-K., Na D.L. (2011). Identification of Pure Subcortical Vascular Dementia Using 11C-Pittsburgh Compound B. Neurology.

[15] Shaw L.M., Vanderstichele H., Knapik-Czajka M., Clark C.M., Aisen P.S., Petersen R.C., Blennow K., Soares H., Simon A., Lewczuk P. (2009). Cerebrospinal Fluid Biomarker Signature in Alzheimer’s Disease Neuroimaging Initiative Subjects. Ann. Neurol..

[16] Hampel H., Buerger K., Zinkowski R., Teipel S.J., Goernitz A., Andreasen N., Sjoegren M., DeBernardis J., Kerkman D., Ishiguro K. (2004). Measurement of Phosphorylated Tau Epitopes in the Differential Diagnosis of Alzheimer Disease: A Comparative Cerebrospinal Fluid Study. Arch. Gen. Psychiatry.

[17] Kaerst L., Kuhlmann A., Wedekind D., Stoeck K., Lange P., Zerr I. (2013). Cerebrospinal Fluid Biomarkers in Alzheimer’s Disease, Vascular Dementia and Ischemic Stroke Patients: A Critical Analysis. J. Neurol..

[18] Schoonenboom N.S.M., Reesink F.E., Verwey N.A., Kester M.I., Teunissen C.E., van de Ven P.M., Pijnenburg Y.A.L., Blankenstein M.A., Rozemuller A.J., Scheltens P. (2012). Cerebrospinal Fluid Markers for Differential Dementia Diagnosis in a Large Memory Clinic Cohort. Neurology.

[19] Román G.C., Tatemichi T.K., Erkinjuntti T., Cummings J.L., Masdeu J.C., Garcia J.H., Amaducci L., Orgogozo J.M., Brun A., Hofman A. (1993). Vascular Dementia: Diagnostic Criteria for Research Studies. Report of the NINDS-AIREN International Workshop. Neurology.

[20] Wardlaw J.M., Smith E.E., Biessels G.J., Cordonnier C., Fazekas F., Frayne R., Lindley R.I., O’Brien J.T., Barkhof F., Benavente O.R. (2013). Neuroimaging Standards for Research into Small Vessel Disease and Its Contribution to Ageing and Neurodegeneration. Lancet Neurol..

[21] Skrobot O.A., O’Brien J., Black S., Chen C., DeCarli C., Erkinjuntti T., Ford G.A., Kalaria R.N., Pantoni L., Pasquier F. (2017). The Vascular Impairment of Cognition Classification Consensus Study. Alzheimers Dement..

[22] Jessen F., Amariglio R.E., van Boxtel M., Breteler M., Ceccaldi M., Chételat G., Dubois B., Dufouil C., Ellis K.A., van der Flier W.M. (2014). A Conceptual Framework for Research on Subjective Cognitive Decline in Preclinical Alzheimer’s Disease. Alzheimers Dement..

[23] Teunissen C.E., Petzold A., Bennett J.L., Berven F.S., Brundin L., Comabella M., Franciotta D., Frederiksen J.L., Fleming J.O., Furlan R. (2009). A Consensus Protocol for the Standardization of Cerebrospinal Fluid Collection and Biobanking. Neurology.

[24] Fazekas F., Chawluk J.B., Alavi A., Hurtig H.I., Zimmerman R.A. (1987). MR Signal Abnormalities at 1.5 T in Alzheimer’s Dementia and Normal Aging. AJR Am. J. Roentgenol..

[25] Duering M., Biessels G.J., Brodtmann A., Chen C., Cordonnier C., de Leeuw F.-E., Debette S., Frayne R., Jouvent E., Rost N.S. (2023). Neuroimaging Standards for Research into Small Vessel Disease-Advances since 2013. Lancet Neurol..

[26] Fasnacht J.S., Wueest A.S., Berres M., Thomann A.E., Krumm S., Gutbrod K., Steiner L.A., Goettel N., Monsch A.U. (2023). Conversion between the Montreal Cognitive Assessment and the Mini-Mental Status Examination. J. Am. Geriatr. Soc..

[27] Smith E.E., Aparicio H.J., Gottesman R.F., Goyal M.S., Greenberg S.M., Schneider J.A., Sorond F.A., Wright C.B., American Heart Association Stroke Council, Council on Cardiovascular and Stroke Nursing (2025). Vascular Contributions to Cognitive Impairment and Dementia in the United States: Prevalence and Incidence: A Scientific Statement from the American Heart Association. Stroke.

[28] Llorens F., Schmitz M., Knipper T., Schmidt C., Lange P., Fischer A., Hermann P., Zerr I. (2017). Cerebrospinal Fluid Biomarkers of Alzheimer’s Disease Show Different but Partially Overlapping Profile Compared to Vascular Dementia. Front. Aging Neurosci..

[29] Eckerström C., Eckerström M., Göthlin M., Molinder A., Jonsson M., Kettunen P., Svensson J., Rolstad S., Wallin A. (2020). Characteristic Biomarker and Cognitive Profile in Incipient Mixed Dementia. J. Alzheimers Dis..

[30] Skillbäck T., Farahmand B.Y., Rosén C., Mattsson N., Nägga K., Kilander L., Religa D., Wimo A., Winblad B., Schott J.M. (2015). Cerebrospinal Fluid Tau and Amyloid-Β1-42 in Patients with Dementia. Brain.

[31] Tsantzali I., Athanasaki A., Boufidou F., Constantinides V.C., Stefanou M.-I., Moschovos C., Zompola C., Paraskevas S.G., Bonakis A., Giannopoulos S. (2024). Cerebrospinal Fluid Classical Biomarker Levels in Mixed vs. Pure A^+^T^+^ (A^+^T_1_^+^) Alzheimer’s Disease. Biomedicines.

[32] Mukaetova-Ladinska E.B., Abdel-All Z., Mugica E.S., Li M., Craggs L.J.L., Oakley A.E., Honer W.G., Kalaria R.N. (2015). Tau Proteins in the Temporal and Frontal Cortices in Patients with Vascular Dementia. J. Neuropathol. Exp. Neurol..

[33] Buerger K., Zinkowski R., Teipel S.J., Tapiola T., Arai H., Blennow K., Andreasen N., Hofmann-Kiefer K., DeBernardis J., Kerkman D. (2002). Differential Diagnosis of Alzheimer Disease with Cerebrospinal Fluid Levels of Tau Protein Phosphorylated at Threonine 231. Arch. Neurol..

[34] Hesse C., Rosengren L., Andreasen N., Davidsson P., Vanderstichele H., Vanmechelen E., Blennow K. (2001). Transient Increase in Total Tau but Not Phospho-Tau in Human Cerebrospinal Fluid after Acute Stroke. Neurosci. Lett..

[35] Mattsson-Carlgren N., Grinberg L.T., Boxer A., Ossenkoppele R., Jonsson M., Seeley W., Ehrenberg A., Spina S., Janelidze S., Rojas-Martinex J. (2022). Cerebrospinal Fluid Biomarkers in Autopsy-Confirmed Alzheimer Disease and Frontotemporal Lobar Degeneration. Neurology.

[36] Pais M., Loureiro J., do Vale V., Radanovic M., Talib L., Stella F., Forlenza O. (2021). Heterogeneity of Cerebrospinal Fluid Biomarkers Profiles in Individuals with Distinct Levels of Cognitive Decline: A Cross-Sectional Study. J. Alzheimers Dis..

[37] Skillbäck T.B., Jönsson L., Skoog I., Blennow K., Eriksdotter M., Zetterberg H., Kern S. (2025). Cerebrospinal Fluid Biomarkers for Alzheimer Disease Among Patients With Dementia. JAMA Neurol..

[38] Wesenhagen K.E.J., Teunissen C.E., Visser P.J., Tijms B.M. (2020). Cerebrospinal Fluid Proteomics and Biological Heterogeneity in Alzheimer’s Disease: A Literature Review. Crit. Rev. Clin. Lab. Sci..

[39] Engelborghs S., Le Bastard N. (2012). The Role of CSF Biomarkers in the Diagnostic Work-up of Mixed Vascular-Degenerative Dementia. J. Neurol. Sci..

[40] Axelsson Andrén E., Kettunen P., Bjerke M., Rolstad S., Zetterberg H., Blennow K., Wallin A., Svensson J. (2023). Diagnostic Performance of Cerebrospinal Fluid Neurofilament Light Chain and Soluble Amyloid-β Protein Precursor β in the Subcortical Small Vessel Type of Dementia. J. Alzheimers Dis..

[41] Mesquita S.D., Ferreira A.C., Falcao A.M., Sousa J.C., Oliveira T.G., Correia-Neves M., Sousa N., Marques F., Palha J.A. (2014). Lipocalin 2 Modulates the Cellular Response to Amyloid Beta. Cell Death Differ..

[42] Li X., Wang X., Guo L., Wu K., Wang L., Rao L., Liu X., Kang C., Jiang B., Li Q. (2023). Association between Lipocalin-2 and Mild Cognitive Impairment or Dementia: A Systematic Review and Meta-Analysis of Population-Based Evidence. Ageing Res. Rev..

[43] Gonzalez-Ortiz F., Kirsebom B.-E., Yakoub Y., Gundersen J.K., Pålhaugen L., Waterloo K., Selnes P., Jarholm J.A., Gísladóttir B., Rongve A. (2025). Associations Between Changes in Levels of Phosphorylated Tau and Severity of Cognitive Impairment in Early Alzheimer Disease. Neurology.

[44] Lewczuk P., Matzen A., Blennow K., Parnetti L., Molinuevo J.L., Eusebi P., Kornhuber J., Morris J.C., Fagan A.M. (2017). Cerebrospinal Fluid Aβ42/40 Corresponds Better than Aβ42 to Amyloid PET in Alzheimer’s Disease. J. Alzheimers Dis..

[45] Xu C., Zhao L., Dong C. (2022). A Review of Application of Aβ42/40 Ratio in Diagnosis and Prognosis of Alzheimer’s Disease. J. Alzheimers Dis..

